# Enhancement of prime editing by recruiting engineered or evolved components and implementing novel strategies

**DOI:** 10.1016/j.bbrep.2026.102495

**Published:** 2026-02-18

**Authors:** Mobina Arabi, Farzaneh Alizadeh, Yasamin Yousefi, Hamed Afarandeh, Sina Mozaffari Jovin, Atieh Eslahi, Majid Mojarrad

**Affiliations:** a Department of Medical Genetics, Faculty of Medicine, Mashhad University of Medical Sciences, Mashhad, Iran; b Medical Genetics Research Center, Faculty of Medicine, Mashhad University of Medical Sciences, Mashhad, Iran; c Genetic Center of Khorasan Razavi, Mashhad, Iran

## Abstract

Prime editing has recently gained attention for its promising potential in treating genetic disorders caused by different types of mutations. This method, based on Clustered Regularly Interspaced Short Palindromic Repeats (CRISPR), has led to the development of new strategies that offer improved editing efficiency. Additionally, the components of prime editing—namely, the prime editor (PE) and the prime editing guide RNA (PegRNA)—have been enhanced through rational design and direct evolution of structural modifications. These improvements have resulted in better performance and new capabilities, driven by novel mutations or components. In this review, we compare various studies that report enhanced versions of PE or PegRNA, which achieve more efficient results. These advancements hold the potential to accelerate and simplify the development of gene therapies for a range of genetic disorders.

## Highlights

• **Optimizing Prime Editing:** Boosting efficiency by modifying Cas9, RT, or PegRNA.
• **Advancing via Design:** Directed evolution & rational design improve editing potential.
• **Epigenetic Modifiers Help:** Enhance efficiency, expanding gene therapy applications.
• **Delivery Challenges:** Efficient targeting remains key for clinical use.

## 1 Introduction

The Clustered Regularly Interspaced Short Palindromic Repeats (CRISPR) system, which functions naturally as the adaptive immune system of bacteria, defends them against invading bacteriophages by destroying their genomes [1,2], and is a widely used tool in genome editing [[3], [4], [5], [6]]. It consists of two main components: a nuclease protein called Cas and a single-guide RNA (sgRNA) that specifies the target on the genome for Cas [7]. The CRISPR system can target RNA, DNA, or even Protein [[8], [9], [10], [11]], depending on the specific Cas protein used. Inside the cell, Cas loading with sgRNA forms an RNP complex that binds to a sequence complementary to the sgRNA and makes a break in the target. This action then activates DNA repair mechanisms, resulting in sequence alterations which can be either intentional or random, depending on the design. SpCas9, a double-stranded DNA-targeting nuclease isolated from *Streptococcus pyogenes*, is the most used Cas protein [12,13] since it has exhibited the highest efficiency among other Cas9 nucleases [14] (see Table 1).

**Table 1:** PE6 variants.

Variant name	RT variant	Preferred RTT	Harbored mutations (engineered/evolved)
PE6a	Evolved Ec48-RT	-	E60K, E279K, and K318E
PE6b	Evolved Tf1-RT	Short less structured	K118R, I128V, K413E, S492 N, P70T, G72V, M102I, and K106R
PE6c	Engineered evolved Tf1-RT	Structured long RTTs	K118R, S118K, I260L, S297Q, and R288Q
			I128V, K413E, S492 N, P70T, G72V, M102I, and K106R
PE6d	Engineered evolved MMLV-RT	Structured long RTTs	D200 N
			T128 N, V129 A/G, P196 S/T/F, N200S/Y, and V223 A/M/L/E

Prime editing is a CRISPR-based gene-editing strategy that typically uses a chimeric protein consisting of a nickase SpCas9 (nSpCas9) harboring the H840A mutation and a MMLV reverse transcriptase (MMLV-RT). This hybrid protein, called prime editor 1 (PE1), is an inefficient PE. The MMLV-RT in the most commonly used prime editor, PE2, is a pentamutant MMLV-RT harboring D200 N, L603W, T330P, T306K, and W313F mutations, with enhanced activity [15]. The prime editing system is guided by an extended form of single-guide RNA, called prime editing guide RNA (PegRNA), that harbors the desired edits to apply to the targeted sequence. The DNA-targeting part of a PegRNA, the spacer RNA, is the same single-guide RNA used in the conventional CRISPR/SpCas9 system, with the same NGG PAM. This spacer RNA is linked to the extension part via a scaffold sequence that includes a primer binding site (PBS) and a reverse transcriptase template (RTT), which introduces the desired edit at the DNA target site. After guiding the PE to the target DNA sequence, the nSpCas9 nicks the PAM-containing strand, creating a free 3՛ end complementary to the PBS region of the PegRNA. This annealed sequence serves as the primer for MMLV-RT reverse transcriptase activity, using the RTT portion of the PegRNA as the template. Once reverse transcription is complete and the prime editing components are gone, there will be two competing flaps to fill the gap in the nicked DNA. If the 3՛ flap wins this competition, the edited DNA will be ligated to the genome, but this is not the end stage. The next challenge for edited DNA to become irreversibly part of the genome is to be selected as the reference strand by the mismatch repair (MMR) system. If not, the opposite strand with an unedited sequence would be considered the reference sequence by the MMR system, leading to reverting the edited sequence to the original undesired one. Unfortunately, because of distinct methylation patterns on the unedited strands, the MMR system mostly uses this sequence as the reference. It reverses the edited strand back to its original sequence. By overcoming this challenge, new generations of PE systems significantly improve the desired editing efficiency, which promises the translation of these tools into clinical applications.

Some inherent advantages make prime editing one of the most promising genome editing approaches. The lack of DNA double-strand breaks (DSBs) makes prime editing safer and reduces the risk of undesired indel formation at on- or off-target sites. The offered expanded editing scope that allows for the correction of almost all kinds of mutations, including all 12 types of point mutations, insertions, and deletions, is another favorable feature of prime editing that makes it outstanding rather than other CRISPR-based gene editing strategies such as base editing, HDR, and conventional CRISPR-mediated editing. In contrast to conventional CRISPR-mediated editing, prime editing can insert or change the specific nucleotides needed, based on the RTT sequence, with a lower risk of unintended genome changes. Furthermore, unlike HDR, prime editing is functional in both dividing and non-dividing cells, making it more applicable to in vivo gene therapy experiments.

Nevertheless, limited insertion or deletion size, and its relatively low editing efficiency can be counted as pitfalls of this promising system. Unexpectedly, the prime editing results are not always precise. Unintended insertion of scaffold sequence is a relatively frequent byproduct of MMLV-RT activity, especially in the case of short RTTs. Therefore, choosing the suitable pegRNA with optimum size of PBS and RTT is an important factor that can impact the accuracy of editing results.

As mentioned above, there are two biased competitions with a lower likelihood of the edited sequence winning. These competitions are among the main explanations for the low efficacy of prime editing, which many studies have sought to address. In addition, PE affinity to the target and its editing activity, as well as pegRNA quality and stability, are among the critical factors that can influence the prime editing rate and efficacy. In this review, we aimed to gather information on more efficient prime editing components or strategies engineered through rational design or evolved through directed evolution.

## 2 Improved prime editing strategies

The first introduced solution to overcome the biased competition between edited and unedited strands for selection as the reference by MMR is to employ a second single-guide RNA to introduce an additional DNA nick approximately 50 nucleotides away from the PegRNA-guided nick on the opposite strand. Such a strategy, named PE3, shifts the game in favor of the edited DNA as the reference for the MMR system, yielding a significantly higher prime editing efficiency (up to 4.2-fold) than PE2. However, this strategy may carry the risk of undesired insertions/deletions (Indels) at the second target site and also increase the risk of off-targeting in the genome [15].

Temporary knockdown of MMR system activity is another way to increase PE efficiency. The PE4 has been developed by combining PE2 with a dominant-negative MLH1 (MLH1dn). This MLH1 variant can bind to the MSH2 component of MMR and impair its function, temporarily inhibiting the MMR system. Additionally, the PE5 strategy, which combines PE4 with a second nicking guide RNA (ngRNA) on the non-edited strand, has shown even greater editing efficiency and more precise results [16]. Taken together, PE4 and PE5 have demonstrated an average 2-fold increase in editing results compared to PE3.

A recent study has introduced an AI-generated MLH1 small binder (MLH1-SB), an 82-amino-acid-long small protein that disrupts the MLHα complex (MLH1 and PMS2) and thereby inhibits the MMR system. MLH1-SB activity is significantly higher than MLH1dn and makes a 29.4-fold and 2.4-fold enhancement in the prime editing results when employed along with PEmax and PE7 (see below), respectively [17].

In addition to MMR activity, P53-dependent cell cycle arrest triggered by Cas9-DNA binding and DNA breakage negatively impacts the genome-editing process, lowering the efficiency of prime editing [[18], [19], [20], [21]]. Transient co-expression of a mouse dominant-negative P53 (mP53DD) or other wild-type P53 suppressors, such as MDM2, has been shown to increase prime editing efficiency by about 2-fold in cells with P53 activity [22]. As expected, PE-Plus, which uses both MLH1dn and P53DD along with PEmax, exhibits the highest efficiency compared with previous approaches [23]. However, it is notable that the expression of a dominant-negative P53 or other P53 suppressors carries the risk of tumorigenesis, posing a safety concern and limiting in vivo applications.

**Fig. 1:**
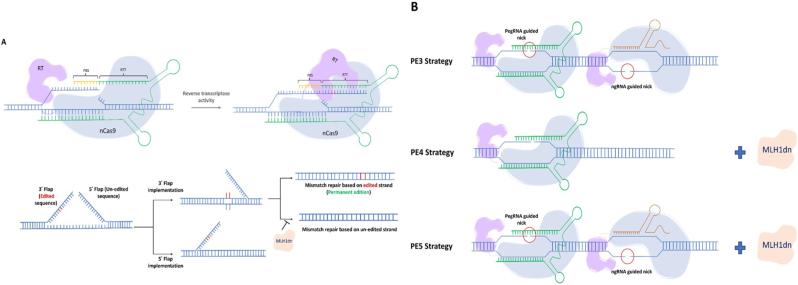
**Prime editing process**. **A**. **Conventional prime editing process**. A hybrid prime editor (PE) protein consisting of a nickase spCas9 and a MMLV-RT nicks 3 nucleotides upstream of the PAM sequence by guidance of the targeting spacer oligonucleotide which is at the 5′ end of the pegRNA. The PBS sequence at the 3′ end of the pegRNA acts as a primer for RT to synthesis the intended edited sequence based on the RTT sequence next to the PBS. Once the newly synthesized oligonucleotide (3′ flap) is completed, it should compete the un-edited flap (5′ flap) for DNA ligation. If the 3′ flap with edited sequence ligate to the DNA, the mismatches caused by implemented edits should then be repaired. If the mismatch repair process uses the edited sequence as the template, prime editing would be successfully done. **B**. **PE3, PE4 and PE5 strategies** that use ngRNA, MLH1dn and both ngRNA and MLH1dn respectively; to prevent MMR system from changing the implemented edits back to the original sequence.

Another recent prime editing strategy with significantly superior efficiency uses wild-type Cas9 with nuclease activity, along with a PegRNA. This approach, which is called PEn, is actually a combination of prime editing and homologous repair (HR), in which applying edits on RTT relies on HR mediated by the homology between RTT and DNA (Fig. 1a). The main drawback of PEn is its high rate of unintended on-target indels, driven by the cell's preference for error-prone NHEJ repair [24]. Using NHEJ blockers or co-expression of NHEJ inhibitors, such as i53 (an inhibitor of 53BP1), can help reduce unintended on-target indels [24,25]. Since using NHEJ blockers has much more safety concerns, co-expression of i53 is the preferred choice and utilized in a PEn strategy called ubiquitin variant-assisted PEn (uPEn) [25]. uPEn has shown up to 8-fold enhancement in applying base conversions compared to PE5max in U2OS cells; however, as expected, this enhancement can be influenced by the type of edits, cell lines and the homology region size, as it enhances when this region size increases up to 15 nt, but plateaus between 15 and 25 nt. uPEn has also demonstrated lower unintended on-target indels than PEn, but remains much less precise than canonical Prime editing [25]. On-target indels aside, PEn and uPEn also exhibit more off-target editing, which heightens their safety concerns and underscores the importance of designing more precise PegRNAs. These approaches are only suitable when high efficiency is the primary goal, on-target indels do not matter (mostly in knock-out approaches), and Cas9 is guided by a precisely designed pegRNA, which minimizes off-target activity [24,25]; however, the off-targeting risk will always be a safety concern.

Among the improved prime editing strategies mentioned, PE3 is the most commonly used option. This preference is likely due to its ease of design and lower safety concerns. In contrast, strategies that utilize an MMR suppressor carry greater risks, as these suppressors can affect the entire MMR system, potentially affecting multiple regions of the genome rather than just the intended target DNA. MMR suppressor strategies, such as PE4, also require an additional protein that must be cloned and included in the delivery vector. This presents a challenge, especially when using adeno-associated viruses (AAVs), as their limited capacity makes it more challenging to deliver the prime editing system.

## 3 Other prime editor variants (non-SpCas9 PEs) expand the editing scope of prime editing

**Fig. 2:**
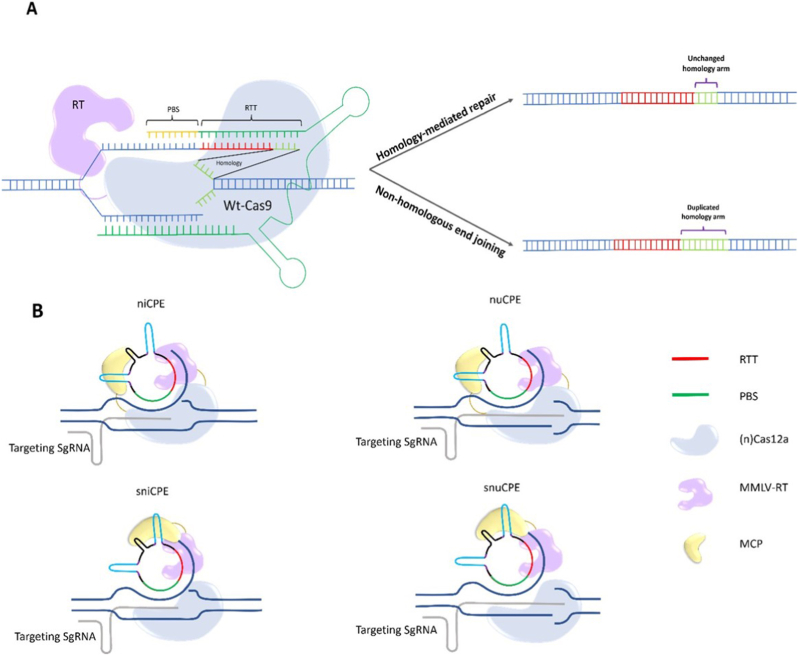
**A. PEn strategy**. Proceeding PEn strategy by homology repair can lead to precise editing; but NHEJ employment results in duplication of homology arm. **B**. Different CPE strategies employing tethered and un-tethered nickase Cas9 (niCPE and sniCPE) or nuclease Cas9 (nuCPE and snuCPE).

G. Liu and colleagues have recently shown the versatile utility of a more compact effector, LbCas12a nickase (nCas12a) in PE structure [26]. Unlike the canonical PEs, this PE variant uses split pegRNA, including an sgRNA and a circular PBS-RTT containing RNA, which provides greater efficiency for nCas12a-PE. Because circular RNA is used as a template, this system is called circular prime editing (CPE) (Fig. 2b). CPE can be used for editing in four different ways: employing tethered nuclease PE (nuCPE), employing split nuclease PE (snuCPE), employing tethered nickase PE (niCPE), and employing split nickase PE (sniCPE). As expected, the precise editing rates are higher in niCPE and sniCPE than in nuCPE and snuCPE. Since the CRISPR-Cas12a system is also a popular tool for multiplex gene editing [[27], [28], [29]], CPE has been evaluated for multiplex prime editing and has successfully achieved quadruple-gene editing using niCPE2 and sniCPE2, with acceptable efficiency (at least 15% at an evaluated locus). To do this, multiple PBS-RTTs would be incorporated into a single circular RNA and delivered along with multiple targeting sgRNAs [26]. Therefore, using CPE may be the preferred choice when the NGG PAM restriction of SpCas9 can be addressed by using LbCas12a, and multiplex gene prime editing is a goal, especially in gene therapies for compound heterozygotes or polygenic diseases. However, targeting multiple sequences may increase concerns about off-target activity. Moreover, the efficiency of this strategy is low (approximately the same as PE2), and future studies should focus on enhancing its efficacy, for example, by engineering or evolving nCas12a to obtain more efficient variants with enhanced activity.

nSaCas9 is another alternative to nSpCas9 for PE, broadening the range of targets due to SaCas9's different specific PAM sequence (NNNGRRT). More flexible mutant SaCas9 (SaCas9 KKH), which recognizes NNNRRT as the PAM sequence [30] is also available for use in prime editing. However, it is important to note that the efficiency of PEs composed of nSaCas9 variants is relatively lower than PEs with nSpCas9, even after improving their nuclear localization [31].

## 4 Enhanced mutant prime editors with improved editing activity

**Fig. 3:**
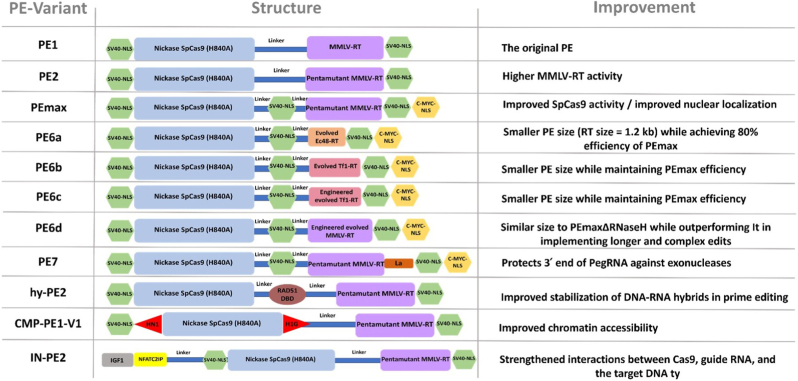
Prime editor variants structures and their improvements**.**

In recent years, new PE protein variants with greater activity have been developed, significantly improving prime editing results (Fig. 3). The first engineered PE2, PEmax, includes a human codon-optimized RT, a 34-amino-acid linker containing a bipartite SV40 NLS, an additional C-terminal c-Myc NLS, and two point mutations, R221K and N394K, in PE2 nSpCas9. This PE variant has demonstrated superior prime editing efficiency (about 2.8-fold) compared to PE2. Since both point mutations are placed in the REC lobe, the enhanced activity of PEmax is probably due to its improved nucleic acid recognition by the REC lobe. Furthermore, combining PEmax with PE3, PE4, and PE5 can yield even more effective prime editing. Implementing PE5max with epegRNAs (see below) has shown increased outcome purity by 4.6-fold in HeLa cells and 3.3-fold in HEK293T cells, indicating further enhancement of editing result, especially in hard-to-transfect cell lines. PEmax's superior efficiency suggests that protein engineering using rational design has the potential to yield enhanced PE variants that pave the way toward achieving the ultimate goal of high-precision prime editing in vivo. Additionally, the effectiveness of codon optimization and nuclear localization on the efficiency of prime editing was clearly demonstrated by PEmax [16].

A recent study hypothesized that the low efficiency of prime editing might be attributed to the low solubility of the Prime editors and the low concentration of available dNTPs in non-dividing cells. Based on this hypothesis, Scot A. Wolfe and colleagues changed the conserved YVDD motif to the YMDD motif in MMLV-RT to transform the onco-retroviral RT active site to a lentiviral-like RT, which has more affinity to dNTPs. The evaluations have demonstrated that this rationally designed PEmax variant has a 2 to 4-fold lower Kd for dNTPs. This prime editor has been confirmed to enhance the prime editing event rate in non-dividing cells, but has no effect in dividing cells that generally have high dNTP concentrations. Subsequently, another point mutations in MMLV-RT were evaluated to identify mutations that can increase the solubility of PEmax. A PEmax variant, PEmax∗∗, was developed that utilizes two point mutations in the MMLV-RT part, L435K and V223 M, which enhance solubility and increase affinity for dNTPs, respectively. This enhanced PEmax variant has shown improved results (about 2-fold) compared to PEmax, underscoring the importance of protein solubility and dNTP use for prime editing efficiency [32].

There are also other solutions to address the dNTP bottleneck in non-dividing cells, such as the transitional suppression of dNTP regulators in targeted cells. SAMHD1 (sterile alpha motif and HD domain 1) is a dNTP-regulator protein found in myeloid cells that decreases dNTP concentrations and inhibits lentiviral infections by impeding their cDNA synthesis. However, the HIV-2-encoded VPX protein can suppress SAMHD1 by ubiquitinating it and promoting proteasomal degradation; therefore, during HIV-2 infection of myeloid cells, elevated dNTP levels enable viral cDNA synthesis and prolong infection [33]. This suppressor protein can be utilized to temporarily elevate dNTP concentration in non-dividing cells targeted for prime editing, thereby enhancing editing outcomes. This approach has demonstrated improved editing results in combination with PEmax, PEmax∗∗, or Twin-PE in non-dividing cells [32]. For future studies, the safety of VPX, a viral-encoded protein that can also have other detrimental effects on cells, especially in vivo, must be evaluated.

The efforts to create compact PEs using wild-type non-MMLV-RTs have not yielded satisfactory results to date. As a result, Liu and colleagues have attempted to engineer more efficient variants of smaller RTs to match the efficiency of MMLV-RT. Despite that, rationally designed compact PEs consisting of non-MMLV-RTs showed significant improvement over their wild types; they were still much less efficient than PE2, except for the engineered Tf1, which showed the same efficiency as PE2; however, it did not succeed in being as efficient as PE2 in implementing longer and more complex RTs. These results indicate that while rational design can sometimes yield more effective variants, it is not always the best approach.

In addition to rational design, directed evolution can also be helpful for identifying more efficient variants through screening many randomly mutated PEs. This approach has yielded even better outcomes than rational design, as there are still unknown characteristics of protein functions and interactions that can be targeted for protein improvement. Subsequent use of phage-assisted evolution on the RT part of PEs for enhancing compact PEs with non-MMLV-RTs has led to the introduction of PE6 variants. These PEs were directed through a positive selection for variants with the capability to restore T7 RNA polymerase expression by reframing the T7 RNA polymerase gene and a negative selection against incapable ones. Different RTs were subjected to this phage-assisted evolution, resulting in different efficient PEs, including PEs consisting of evolved *Escherichia coli* Ec48 retron RT (evo-Ec48), named PE6a, and evolved *Schizosaccharomyces pombe* Tf1 retrotransposon RT (evo-Tf1), named PE6b. Between these evo-RTs, evo-Tf1 has demonstrated the best function, comparable to PEmax, but evo-Ec48 is the smallest (1.2 kb vs. 2.1 kb for MMLV-RT) and achieves 80% efficiency of PEmax, making it the preferred choice when a highly minimized PE is needed. Additionally, to enhance the efficiency and productivity of evo-Tf1, rational engineering was also employed to introduce point mutations that had previously shown improved results. The PE6c variant that utilizes this engineered-evolved-Tf1 (eng-evo-Tf1) has demonstrated greater efficiency in applying longer, more complex edits than PE6b and PEmaxΔRNaseH (a version of PEmax in which the RNaseH domain of RT is removed). Similarly, the PE6d variant, which consists of an engineered-evolved-MMLV-RT (eng-evo-MMLV-RT) with a similar size to PEmaxΔRNaseH, has also outperformed PEmaxΔRNaseH in implementing longer and complex edits as it can compensate for the lack of an RNaseH domain by maintaining a small RT size. As an interesting coincidence, one of the random mutations found in the evolved MMLV-RT during phage-assisted evolution that makes it more efficient is Q492X, which retains the intact polymerase domain and removes the RNaseH domain, resulting in a similar structure to PEmaxΔRNaseH. Other positively evolved mutations are also placed just in the polymerase domain, indicating the importance of this domain and the negligible effect of the RNaseH domain on the prime editing process. Another mutation that coincidentally overlaps with another enhanced PE variant is V223 M, which was also rationally designed to develop a PEmax variant with higher affinity to dNTPs. This coincidence demonstrates the effectiveness of directed evolution in obtaining efficient variants, even those previously hypothesized or proven to be more efficient [34].

The latest assessments of PE6 variants have shown that less processive RTs, such as those in PE6b and PEmaxΔRNaseH, have a lower edit/indel ratio when used alongside longer, structured RTTs or in paired pegRNA strategies such as Twine-PE, because they prematurely stop during polymerase activity. However, these RTs are preferred for applying edits from short, unstructured RTTs. The more processive RTs implement the scaffold sequence in addition to RT and therefore would have a lower edit/indel ratio. These more processive RTs are suitable for implementing long edits from structured RTs or working with paired pegRNAs systems.

Each PE6a-d variant offers specific advantages and disadvantages that can be used depending on the RT length, complexity, and the employed strategy. PE6a offers the most efficient small PE, making it the best choice for delivery strategies that require highly minimized PEs, while PE6b offers an efficient PE to use alongside short, less complex RTTs with higher free energies. PE6c, which is a more processive version of PE6b, has an improved ability to apply edits from longer, more complex RTTs or work properly in paired pegRNA strategies, and eventually, PE6d, which benefits from using eng-evo-MMLV-RT, also offers the advantage of efficient performance, particularly in structured, long RTTs or in more complex strategies such as Twine-PE.

In addition to engineered and evolved RTs, the nSpCas9 also acquired some mutations that enhanced its activity. In contrast to RTs, enhancing mutations in nSpCas9 are not clustered in a specific domain. These data suggest that all domains of nSpCas9 are effective in its activity. PE6 variants harboring nSpCas9-enhancing mutations are labeled as PE6e-g, and all of them have shown improved activity [34].

Another evolutionarily directed PE variant (PE-Y18), which exceeds the efficiency of PEmax, has also been introduced through a yeast-based directed evolution system called OrthoRep. PE-Y18 shows substantially exceeded activity than PEmax in both truncated (PE-Y18ΔRnH) and non-truncated forms, in vivo and in vitro, with no elevated off-target activity [35]. This enhanced PE variant harbors two point mutations: one in the REC lobe of nSpCas9 (A259D) and another in the connection domain of MMLV-RT (K445T). Since the in vitro evaluation of PE-Y18 showed no increase in DNA nicking or reverse transcription activity, the enhanced editing outcomes are likely due to improved priming of the PB [35], stronger interactions between PE and nucleic acids, or improved target detection by PE.

Since the employment of free single-stranded DNA (ssDNA) by RT as a primer is an essential step for prime editing, stabilizing ssDNA and the PBS hybrid could be beneficial. To do this, an ssDNA-binding domain (ssDBD) can be added. Rad51 is a conserved protein that plays a role in homologous repair of DSBs by binding to ssDNA, facilitating the search and invasion of homologous DNA sequences [36,37]. It can also bind to single-stranded RNAs, making it a good choice for facilitating and stabilizing DNA-RNA hybrids in prime editing [38]. The hyPE2, which contains a Rad51 protein domain between nSpCas9 and MMLV-RT domains, has shown higher efficiency (1.4-1.5-fold) than PE2 in editing different targets using various pegRNAs; however, it has also demonstrated a slightly higher level of unintended edits, such as indels [38].

Moreover, hyPE2 has only been evaluated in cells with lentivirally integrated PegRNAs, and this integration could affect the results. So, it is important to assess this recombinant PE2 variant further using non-integrating approaches.

Generally, interactions between Cas9, the guide RNA, and the target DNA are important factors that significantly affect CRISPR/Cas9 editing efficiency; therefore, fusing peptide domains that strengthen these interactions to the Cas9 protein can enhance outcomes. This theory has been validated by a PE2 version, which incorporates two fused 85-amino acid peptides that were selected from a library of 12000 peptides fused to nSpCas9 based on their ability to improve prime editing efficiency. These peptides include a phosphomimetic IGF1 peptide and an NFATC2IP peptide (IN-PE2), resulting in a 60% increase in editing efficiency [39].

Recently, a new PE7 variant has been introduced, showing a protective role for the 3′ end of pegRNA against exonucleases. This variant carries a protective truncated La protein linked to the C-terminus of PE, which protects pegRNA by binding to its polyuridine tail transcribed by RNA pol III at the 3′ end of pegRNA. Reported results have demonstrated a median of 21.2-fold and 5.5-fold enhancement rather than PE2 when using PE7 along with pegRNA and epegRNA (see below) in U2OS cells, respectively [40,41]. PE has been modified in many ways to boost its efficiency. So far, each change has been tested separately, but combining them might yield even better results.

## 5 Improved chromatin accessibility results in more efficient prime editing

Predicting the efficiency of CRISPR/Cas9 strategies has always been challenging due to the variation between cell types and different loci. Chromatin accessibility is among the most important reasons why the same strategy and design may not work equally well across different cell types; therefore, overcoming this hindrance can improve Cas9 access to DNA and, consequently, increase CRISPR/Cas9 efficiency [[42], [43], [44]]. A potential solution to overcome heterochromatin's impeding effect on Cas9's access to DNA is to use a transcription factor along with Cas9 to loosen nucleosomes and the chromatin structure [45]. P65 is a transcription factor whose functional chromatin loosening has been proven during its utilization in a prime editing strategy with the Suntag system. In this approach, GCN4 peptides are attached to the PE, while P65 is linked to single-chain variable fragment (ScFv) antibodies, guiding the transcription factor to the targeted site by binding to the GCN4 peptides. This approach has demonstrated improved efficiency in combination with PE2 (average 1.65-fold), PE4 (average 1.55-fold), and PE5, compared to the controls [46]. CMP-PE-V1 is another PE variant with improved chromatin accessibility, consisting of chromatin-modulating peptides (CMPs) at both terminals of nSpCas9, including high-mobility group nucleosome binding domain 1 (HN1) at the N- terminus and a histone H1 central globular domain (H1G) at the C-terminus. CMP-PE3-V1, which utilizes an ngRNA, demonstrates significantly higher efficiency (up to 3.9-fold) compared to PE3 [47]. These successful studies confirm the significance of chromatin structure in PE's access to DNA. However, given the greater accessibility of the genome to PE, this approach might also increase off-target effects, raising safety concerns about its use. Especially if chromatin accessibility increases with PEn use, it is predicted to mediate even more off-target indels.

## 6 Engineered pegRNAs with improved prime editing efficacy

**Fig. 4:**
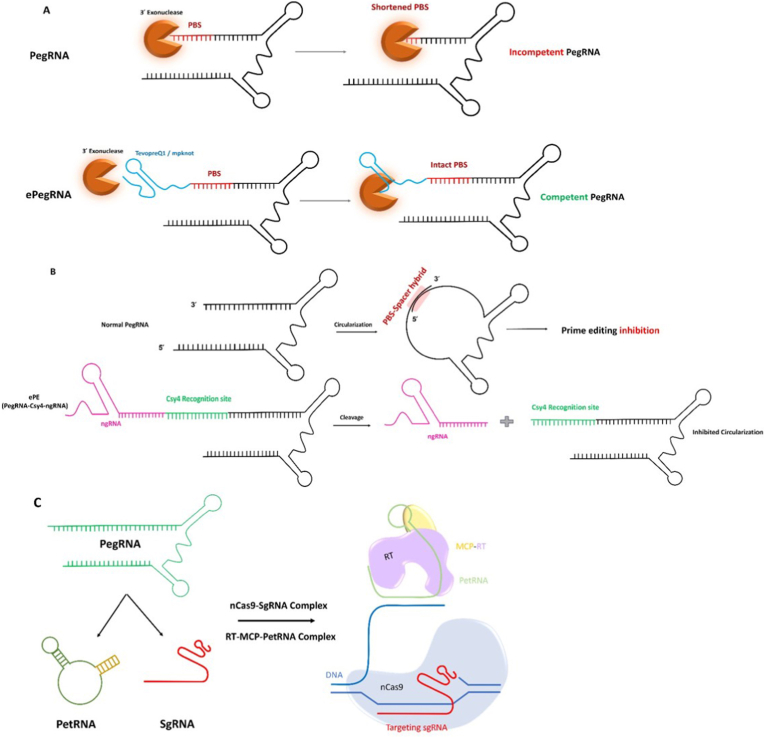
**A. ePegRNA protection against 3՛ exonucleases.** Presence of mpknot or TevopreQ1 aptamer at the 3՛ end of pegRNA protect it from degradation and formation of incompetent pegRNAs by exonucleases in the cells. **B. ePE.** ePE benefits from a Csy4 recognition site prevents circularization of PegRNAs and decreasing the prime editing efficiency. **C. Split-PE (sPE).** nCas9 reaches the target DNA by sgRNA guidance and then MCP-RT starts its transcriptase activity by using petRNA (containing PBS-RTT sequences) as the primer and template sequence.

PegRNAs are sensitive to cellular 3′ exonucleases, which may lead to partial degradation of PBS and RTT sequences, resulting in inefficient prime editing due to the aggregation of incompetent, deficient pegRNAs with impaired PBS and RTT in cells, competing with competent intact pegRNAs. The first introduced solution to address this problem has led to the development of engineered pegRNAs (epegRNAs), which harbor either a tevopreQ1 (a trimmed modified prequeosine1-1 riboswitch aptamer) or an mpknot (the frameshifting pseudoknot from Moloney murine leukemia virus) RNA motif at the 3′ end of pegRNA, protecting the integrity of PBS and RTT sequences (Fig. 4a). epegRNAs have been able to improve prime editing efficiency 3 to 4-fold in different cell lines, demonstrating their wide application range. However, using epegRNAs comes with complications, such as designing an eight-nucleotide linker RNA between the PBS and RNA motif to prevent undesirable secondary structure in the PegRNA, which can reduce prime editing outcomes. To address this problem, the pegLIT algorithm (https://peglit.liugroup.us/) has been developed to suggest suitable RNA linkers specific to each spacer and extension sequence of each pegRNA, with the lowest chance of incorporation into an undesired structure [48]. Similarly, Xr-PegRNA containing a Zikavirus exoribonuclease-resistant RNA motif and G-PE, carrying a G-quadraplex structure at the 3′ end of pegRNAs, reduces exonuclease-mediated degradation and therefore, increases competent pegRNAs for prime editing [49,50].

In addition to pegRNA degradation in cells, the potential circularization of pegRNA due to homology between the PBS and spacer oligonucleotides hampers optimal prime editing activity. Two new methods, ePE and tethered PE (tPE), that use non-circularizable pegRNAs have been developed to address this issue. ePE uses a pegRNA with a fused 20-nt Csy4 cleavable recognition site, which remains a hairpin structure at the 3′ end of pegRNA and releases the ngRNA (Fig. 4b). The tPE method is based on tethering the pegRNA to the PE-MCP hybrid protein via an MS2 aptamer. ePE and tPE potentially inhibit pegRNA circularization by extending and stabilizing the 3′ terminus of pegRNAs, leading to an increase in the desired editing outcomes up to 5-fold and up to 3.7-fold, respectively, depending on the genomic locus and cell type.

Despite the detrimental effect of circularized pegRNAs subsequent to PBS and spacer oligonucleotides base pairing, a circular pegRNA with ligated ends could be more stable. Split PE (sPE), which employs nCas9 and RT separately, utilizes split pegRNAs, in which the targeting spacer oligonucleotide and PBS-RTT are separately delivered to the target site [51] (Fig. 4c). In this strategy, the targeting guide RNA (SgRNA) guides nCas9 to the target sequence, and PetRNA, an MS2-containing circular RNA harboring PBS-RTT, binds to MCP-RT, providing a primer for its reverse transcriptase activity. Evaluations have supported the idea that petRNA exhibits non-effector-dependent stability due to its circular structure, whereas pegRNA requires the Cas9 effector for stabilization in cells. This circular petRNA exhibits the same efficiency as pegRNA in canonical prime editing, unlike linear petRNA, which has shown lower efficiency [51]. Moreover, safety should be considered when evaluating specific methods in vivo. Specifically, the stability of engineered pegRNAs and circular RNAs, such as petRNA, may stimulate innate immune responses [[52], [53], [54]]; therefore, it is necessary to evaluate them in the context of immunity in future research. In addition to engineering pegRNA itself, the promoter that controls pegRNA expression can also be changed to employ RNA Polymerase II instead of RNA Polymerase III. This can help address transcription termination that can occur when RNA Polymerase III transcribes poly(T)-containing pegRNAs. This prime editing system, named p2PE3, has shown equal efficiency as ePE when not using poly(T)-containing pegRNAs in prime editing, but it outperforms ePE (13.3-fold) when a poly(T)-containing pegRNA should be transcribed. This system uses the herpes simplex virus 1 (HSV-1) latency-associated intron to retain the functional gRNA in the nucleus and safeguard it from degradation. Generally, RNA Pol II promoters are preferred for editing T-rich target sequences or when a controllable promoter is needed, whereas RNA Pol III promoters are constitutively expressed [55].

Generally, efforts to enhance pegRNA efficiency primarily focus on stabilizing the pegRNA structure to maintain its integrity within cells. The ultimate goal is to design stable pegRNAs, as this is essential for achieving higher efficiencies with the prime editing system. It is also predictable that using enhanced stable pegRNAs in combination with improved PEs may yield results that surpass the efficiency achieved with either approach alone.

## 7 PegRNA design: The key player in prime editing

PegRNA design is an important step in conducting a prime editing project, as a poorly designed pegRNA may not yield desirable prime editing efficiency even in the presence of the most efficient PEs. Given the importance of this fundamental step, some studies have developed algorithms to predict desirable and undesirable features in each pegRNA [56,57], which are intended to implement nucleotide substitutions, small insertions, or deletions. PegRNA design involves variable factors for each target sequence, such as the PAM location, the edit position, and the PBS and RTT sequences. PBS and RTT lengths are important factors with a high impact on prime editing results. If the GC content of the PBS is lower than 40% and the GC content of the RTT is higher than 60%, it is recommended to use longer PBS and shorter RTT. This is because the stronger hybridization of PBS to the 5′ end of the nicked DNA, combined with the weaker stability of RTT-cDNA, is beneficial. However, GC content of RTT, rather than RTT length, is not among the 40 top-ranked factors affecting prime editing efficiency [56]. In addition to PBS and RTT lengths, the features related to their nucleotide sequences, such as melting temperature, also impact the pegRNA-related prime editing efficiency (summarized in the table?). Other than PBS-RTT, the spacer oligonucleotide, which determines the targeted sequence, also impacts the prime editing efficiency, as the DeepSpCas9 score (computationally predicted Cas9 nuclease activities at a given target sequence [58]) has the most impact on prime editing efficiency (a higher DeepSpCas9 score is favorable) [56]. These considerations are proven to be related to pegRNA folding, as the undesirable structured folding of pegRNA due to hybridization of spacer-PBS has been demonstrated to impact the efficiency of prime editing negatively. The higher Tm of spacer-PBS hybrids make the Cas9-loading harder and therefore, the 3′ truncated pegRNAs with shorter PBS and lower Tm of spacer-PBS hybrid strongly compete with intact pegRNAs for Cas9-loading and make the intended prime editing less efficient. This parameter should be considered separately for in vivo (delivering a coding plasmid into cells) and in vitro (delivering mRNA or RNP) complex formation, as the predicted folding of pegRNA differs in these contexts. For example, if pegRNA is folding in vitro, shorter PBS lengths are more efficient for Cas9 loading. In contrast, if it is folding in vivo, shorter PBS lengths are not preferred, as hybridization stability is lower in vivo than in vitro [59].

Generally, the efficiency of prime editing varies across edit types (insertions, deletions, and substitutions); while insertions have the highest efficiency, substitutions have the lowest. Moreover, not only does the edition type matter, but the specific features of the intended edits affect the efficiency of the results. The 1 to 2 bp insertions or deletions are similarly efficient, but exhibit reduced results as the size of insertions or deletions increases. Specific features of substitution editing also impact the efficiency of prime editing. The most efficient substitution implemented by PE2 is the C-to-T transition, and the lowest substitution rates are observed when the T-to-G transversion is intended [56]. PAM disrupting synonymous mutations co-implementing with the desired mutation can dramatically increase on-target editing efficiency and decrease on-target unintended indels. By doing this, PAM would disappear after successful editing; therefore, the PE would not attach to the target site repeatedly, and could not induce indels over subsequent targeting. Implementing silent same-sense mutations using sPegRNAs has also been shown to increase on-target intended editing by confusing the MMR system and reducing the likelihood of reverting to the original sequence. Combining spegRNA with apegRNA, which harbors a base substitution in the small hairpin of scaffold (apegRNA) in addition to the same-sense mutation (spegRNA), the prime editing results can be even more enhanced [60].

In conclusion, pegRNA design should be precise and careful, given its importance to the outcomes of prime editing. Many factors impact pegRNA efficiency, and it is generally challenging to address all of them; therefore, it is recommended to focus on the more important ones if a pegRNA targeting a specific sequence cannot be designed perfectly. Several online tools have been developed to design pegRNAs with high efficiency and specificity. These tools include PrimeDesign [61], PE-designer and PE-analyzer [62], Easy-prime [63], Peg-IT [64], DeepPrime [65] and SynDesign [66]. Each tool offers different options for predicting pegRNA efficiency based on its features. To ensure that the designed pegRNAs are effective, properly folded, and specific to the on-target with the lowest predicted off-target editing, we strongly recommend qualifying the pegRNAs using at least three pegRNA designer tools (see Table 2).

### 7.1 Novel strategies to improve large insertions or deletions resultsoved chromatin accessibility results in more ef

**Fig. 5:**
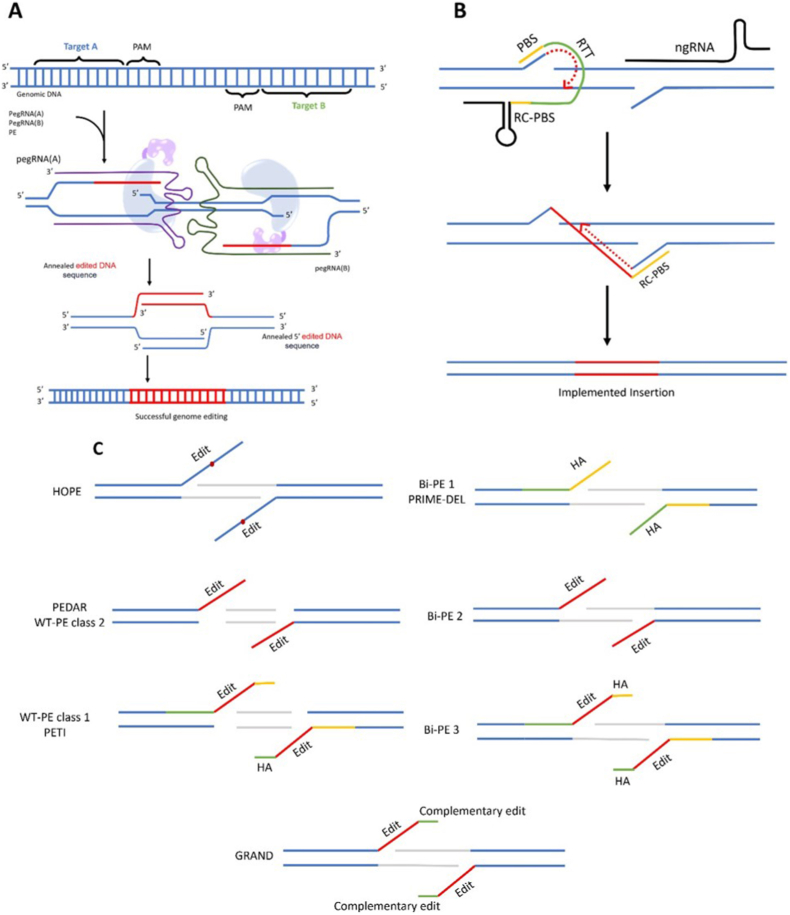
**Twin-PE mechanism of action.** Two pegRNAs with two different PAM and direction but complementary RTTs should be designed and delivered into the cells. Once the 3′ flaps with edited sequences are released, hybridization of 3′ flaps and 5′ flaps occur. If the 3′ flaps hybrid win the ligation competition, the prime editing is successfully done. **B. TJ-PE mechanism of action.** The TJ-PE specific pegRNA contains two types of priming sites: one is the traditional priming site (PBS) located at the 3′ end of the pegRNA, and the other is a reverse complement PBS (RC-PBS). The RC-PBS is also utilized as a RT template next to the RTT. Once reverse transcription is complete, the RC-PBS hybridizes with the nicked DNA adjacent to the last nicked sequence and serves as a primer to synthesize the complementary strand for lastly synthesized sequence. **C. RTT reverse transcription results of different paired pegRNA strategies.**

Since conventional prime editing with a single pegRNA is inefficient for large insertions, deletions, or replacements, several novel strategies have been developed to overcome this limitation while retaining a prime-editing approach (Fig. 5c). All of these strategies use paired pegRNAs with complementary RTTs (partially or completely) [[67], [68], [69], [70], [71], [72], [73]] and some of them also have a homology arm to the 5′ original nicked sequence of another pegRNA [[70], [71], [72], [73]]. These strategies result in a lower indel/edit ratio than the Cas9/double-sgRNA strategy for making large deletions; therefore, they may be preferred for precise, in-frame deletion (summarized in Table 3). Twin-PE was the first paired pegRNA method introduced and showed superior efficiency for implementing larger insertions up to 113 nucleotides [67,76]. This method involves two pegRNAs, each targeting an opposite DNA strand (Fig. 5a). The RTT sequences of these pegRNAs must be completely or mostly complementary, so the newly synthesized strands would hybridize due to their complementary sequences. The resulting hybrid of the two synthesized strands improves the chances of their implementation in the genome, as there would be no mismatch problem. Because no mismatch is formed in the edited sequence, the MMR system would no longer be involved, preventing the edited sequence from reverting to the unedited sequence. Other paired pegRNA methods are mostly based on the Twine-PE mechanism of action but differ in some details. Some of these methods, such as Bi-PE [73] and PRIME-Del [71], utilize homology arms (HA) that are complementary to surrounding target sequences, increasing the likelihood of integration into the genome for the hybridized edited strands. Some other methods, including PEDAR [69] and WT-PE [70], use wild-type Cas9 nuclease to reduce the risk of reintegration of unedited hybrid sequences. Recently, a novel strategy, template-jumping prime editing (TJ-PE) [75] has been introduced, which uses a single, specifically designed pegRNA and an ngRNA to insert the desired sequence into the genome (Fig. 5b). This approach mimics the insertion mechanism used by non-LTR retrotransposons. It involves using a pegRNA consisting of two PBS sequences instead of one. The first PBS sequence plays the formal role, while the second is a reverse complement PBS (RC-PBS2) placed after the RTT sequence at the 3′ end of the pegRNA. Once the first PBS anneals to the nicked sequence (guided by the pegRNA), RT synthesizes the RTT complementary DNA. This is followed by the synthesis of RC-PBS2 complementary DNA, which hybridizes with the nicked sequence (guided by ngRNA) on the opposite strand. This second PBS hybridization provides another primer for complementary elongation of the newly synthesized DNA, leading to the formation of hybridized sequences that may integrate into the genome.

**Table 2:** Top-10-ranked variable factors with highest impact on prime editing results [56].

Rank	Variable factor	Favorable mode
1	DeepSpCas9 score	Higher score
2	GC count in PBS	Higher numbers (up to 60% GC content)
3	Tm of PBS	Up to 35 ֯ C
4	Number of UU in the extension part of pegRNA	Lower numbers
5	Tm of target DNA region corresponding to the RTT	Up to 35 ֯ C
6	T at position 16 in the wide target sequence	Absence
7	Length of PBS + RTT template region	Shorter lengths
8	C at position 17 in the wide target sequence	Presence
9	Length of the RTT	Shorter lengths
10	G at position 24 in the wide target sequence	Absence

**Table 3:** Novel paired pegRNA strategies.

Strategy	Homology arm to the original DNA	PegRNA design	Explanations
Twin-PE [67]	No	Two opposite-directed pegRNAs with completely complementary RTTs	Deletions less than 1 kb Insertions up to about 100 bp
HOPE [74]	No	Two opposite-directed pegRNAs with completely complementary RTTs	Paired pegRNAs encode the same edits in both sense and antisense DNA strands
Bi-PE [73]	Yes	Two opposite-directed pegRNAs with (insertion)/without (deletion) partially complementary RTTs + HA	Deletions up to about 650 bp Insertions up to about 20 bp
Prime-Del [71]	Yes	Two opposite-directed pegRNAs with partially complementary RTTs + HA	Deletions up to 10 kb Insertions up to about 100 bp
GRAND [68]	No	Two opposite-directed pegRNAs with partially complementary RTTs	No homology between RTT and deleted sequence should exist/Deletions up to up to about 1.3 kb Insertions up to about 1 kb (most efficient for less than 400 bp)
PEDAR [69]	No	Two opposite-directed pegRNAs with completely complementary RTTs + nuclease Cas9	Deletions up to 10 kb Insertions up to 60 bp
Bi-WT-PE class 1 (70)	No	Two opposite-directed pegRNAs with completely complementary RTTs + nuclease Cas9	Deletions up to Mb scales
Bi-WT-PE class 2 (70)	Yes	Two opposite-directed pegRNAs with partially complementary RTTs + HA	Deletions up to Mb scales
TJ-PE [75]	Yes (RC-PBS2)	One pegRNAs with two PBS + a nicking gRNA	The 3′ extension of TJ-pegRNA should contain an insertion sequence, PBS 1 and a reverse complement sequence of PBS2, complementary to nicked sequence by ngRNA (RC-PBS2)/No homology between RTT and deleted sequence should exist/insertion up to about 800 bp Deletion up to 90 bp
PETI [72]	Yes	Two pegRNAs with HA as RTTs	Two pegRNAs generate HA compatible to another genomic location/induce translocations and inversions

The prime editor–mediated correction of nucleotide repeat expansion (PE-CORE) [77] has recently been reported to effectively shorten triple nucleotide expansions, using the exact mechanism as paired pegRNA methods. The best results were achieved when homology arms were used, and the triple nucleotides were replaced with the same-sense codons. This reduces the chance of hybridization between newly synthesized strands and long unedited strands with the exact complementary sequence. This approach is effective in correcting nucleotide repeat expansion in HEK293T cells and in iPSCs derived from SBMA and SCA1 patients.

In addition to the mentioned paired pegRNA methods (which are mostly more flexible for deletions than insertions), there are other approaches that combine prime editing with Bxb1 serin integrase to insert larger sequences into the genome [76]. As an example, Twine-PE could be designed to insert the Bxb1 integrase attB substrate sequence [78]. After successful insertion of the attB sequence into the genome, the next step is to introduce the donor gene next to an attP sequence, along with a Bxb1 integrase-coding plasmid, to mediate integration of a larger donor gene into the genome. Another method, called Programmable Addition via Site-Specific Targeting Elements (PASTE) [79], uses a variant of the PE system consisting of a Bxb1 element linked to the standard PE to insert large intended sequences. In this method, after adding the attB sequence using PE3, the donor gene next to an attP sequence is inserted into the attB site using Bxb1-PE integration.

In conclusion, the challenge of inserting or deleting large sequences has been addressed through the introduction of various paired pegRNA strategies, which have enhanced prime editing's capabilities for executing larger edits.

## 8 Transfection or AAV transduction rates have a significant impact on editing efficacy

There are multiple factors affecting prime editing efficiency beyond PE functionality and the quality of pegRNA design, such as cell transfection rate, which determines the amount of plasmid entering cells. An improved entry of coding plasmids for prime editing components leads to more efficient prime editing. This is another reason why the same strategy and the same pegRNA design yield more efficient results in easy-to-transfect cell lines than in hard-to-transfect ones. Conventionally, two (PE2) or three (PE3) plasmids are co-transfected into cells to carry out prime editing projects. Co-transfection of multiple plasmids may be less efficient. Therefore, using a single vector containing all prime editing components could improve efficiency. A recent study has supported this idea by creating all-in-one PE3 and PE5 plasmids, which resulted in 4-fold and 5.7-fold improved efficiency, respectively, compared to using multiple plasmids for transfection [80].

Employing prime editing in vivo is limited due to the AAV genome packaging capacity (about 4.7 Kb) [81] and the large size of PE2 (6.3 Kb). To overcome this limitation, the common solution is to split the PE coding gene into two parts and package them in two different AAVs, using inteins at the C and N-termini of each part of PE. These two parts of the PE would then be attached during intein-mediated protein ligation within the cells; however, it will never be as efficient as delivering the full-length PE. The dual AAV approach has been widely used in various studies and has shown promising results in animal models [[82], [83], [84], [85], [86], [87]]. Intein split processing of PE is dependent on the position of the intein split site. A study compared PEs with commonly used Cas9 split sites (573-574, 637-638, 674-675, and 713-714) and two types of inteins (Npu and Rma) to evaluate their efficiency. This study found that intein processing mediated by Rma (573-574) and Rma (674-675) showed the highest efficiencies when using intein-harboring plasmids. However, due to the C-terminal being about 5 Kb in size, which exceeds the packaging capacity of AAV, the use of these split sites is limited to truncated PE delivery by AAV, such as PECO-Mini, which has been used in the same study and consists of a trimmed nSpCas9 and a ΔRNaseH MMLV-RT [88].

Intein processing can pose a potential bottleneck, reducing the efficiency of the prime editing process. Moreover, the requirement to deliver two AAVs into target cells in vivo raises concerns about the increased dose of virus particles that must be systematically injected, potentially leading to safety concerns about immunological reactions post-injection. A study has shown that using nSpCas9 and MMLV-RT-MCP separately, without any linkage between them, known as split PE (sPE), also demonstrates equal efficiency to PE2 when used with a circular petRNA (containing the PBS and RTT parts linked to the MS2 aptamer) and sgRNA [51]. Consistent with these results, another study hypothesized that the MMLV-RT might not work in cis with nSpCas9, but might work in trans with another nSpCas9. They evaluated a split PE without using the MCP and MS2 aptamer, and the results were promising. They found that using nSpCas9, MMLV-RT, and an intact pegRNA could be as efficient as canonical PE2, but more efficient for viral delivery [89]. Using untethered PE has the advantage of intein-independent AAV packaging of each Cas9 and RT, eliminating the complex process of protein assembly via intein splicing and leading to more efficient prime editing.

Another potential solution to the capacity issue in PE delivery is to use new, compact PEs that can be packaged into a single AAV. To date, related efforts have not achieved success. The first efforts to minimize PE size were based on the MMLV-RT truncation, since the RNase H domain is not necessary for implementing the intended edits. RNase H activity is not only important for prime editing but also has been shown to interact with eRF1, an essential factor in terminating protein translation [90,91]. This interaction has the potential to be harmful for translation termination and cause stop-codon readthrough [92]. Based on this knowledge, research has been conducted to evaluate the efficiency of PE variants consisting of a truncated MMLV-RT (ΔRNaseH) and showed that RNase H removal does not affect PE activity [88,89,93]; In fact, removing RNase H increases lentiviral production yield as the packaging size decreases [88]. However, even after RNAse H removal, PE size still does not rely on the packaging capacity of AAV. Recently, Mini-PE [94] has been reported as a compact PE consisting of *Campylobacter jejuni* Cas9 (CjCas9) and a truncated MMLV-RT, with a total size of 4.5 Kb, which relies on AAV packaging capacity. However, Mini-PE exhibited an editing efficiency below 1% in vivo. In the future, other types of PEs consisting of different Cas9 proteins fused to different types of RTs might be introduced with comparable editing efficiency to PE2.

## 9 In vivo studies confirm the effectiveness and accuracy of prime editing

Numerous studies have reported successful prime editing using various types of PE, PegRNAs, and strategies, primarily employing adeno-associated viruses (AAV) as delivery vectors. For instance, Gao and colleagues [95] conducted a comparison of in vivo editing in mouse zygote genomes, contrasting the homology-directed repair (HDR) method with prime editing (PE2). They found an editing efficiency of 55.65% using HDR compared to 20.74% with the prime editing method, which was specifically designed to implement a base substitution in the CArG box, a regulatory region of the Tspan2 gene. Despite the lower on-target efficiency, the prime editing results were more accurate, showing no off-target activity, while HDR-edited mice demonstrated a 40.11% rate of undesired indels.

Another study utilized prime editing to target the Dnmt1 and Pah genes in the mouse liver genome, both in vitro and in vivo [96]. In vitro assessments were conducted to evaluate the potential of various PegRNAs for gene editing, allowing researchers to select the most effective ones for in vivo use. They compared the editing results obtained using AAV-delivered intein-split PE and adenovirus (AdV)-delivered unsplit PE2ΔRNaseH. The study reported a prime editing efficiency of 14.4% with AAV as the delivery vector and 58.2% with AdV. The efficiency of editing the Pah gene was consistent with that of the Dnmt1 gene. In neonate mice injected with AAV vectors co-expressing intein-split PE2ΔRNaseH and PegRNA, very low editing efficiency was observed (greater than 2%). In contrast, delivering an AdV vector encoding the unsplit PE2ΔRNaseH and PegRNA resulted in an editing efficiency of 6.9%. By adding an additional guide RNA (PE3), the efficiency increased to 11.1% (a 60% improvement) without any detectable off-target effects.

Other studies have also highlighted the potential of prime editing in treating genetic disorders such as sickle cell anemia [97] and Leber congenital amaurosis [86], again demonstrating no detectable off-target activity through in vivo prime editing experiments. These promising findings suggest that prime editing could eventually be tested in future clinical trials and may lead to approved gene therapies. Low off-target effect and high precision are among the main properties that make prime editing an attractive choice for in vivo genome editing. promising results of successfully genome edited mice with cured genetic disorders such as sickle cell anemia, phenylketonuria, Duchenne muscular dystrophy and other disorders along with approximately no off-target effect shows that this genome editing tool has the potential to be tested in future clinical trials and hopefully future approved gene therapies.

## CRediT authorship contribution statement

**Mobina Arabi:** Conceptualization, Writing – original draft. **Farzaneh Alizadeh:** Writing – original draft, Writing – review & editing. **Yasamin Yousefi:** Visualization, Writing – original draft. **Hamed Afarandeh:** Visualization, Writing – review & editing. **Sina Mozaffari Jovin:** Conceptualization. **Atieh Eslahi:** Supervision, Writing – original draft, Writing – review & editing. **Majid Mojarrad:** Conceptualization, Supervision, Validation, Writing – original draft, Writing – review & editing.

## Declaration of competing interest

The authors declare that they have no known competing financial interests or personal relationships that could have appeared to influence the work reported in this paper.

The work described has not been published previously.

The article is not under consideration for publication elsewhere.

The article's publication is approved by all authors and tacitly or explicitly by the responsible authorities where the work was carried out.

## Data Availability

No data was used for the research described in the article.
